# A scoping survey for the UK rheumatology occupational therapy capabilities framework

**DOI:** 10.1093/rap/rkaf072

**Published:** 2025-06-09

**Authors:** Yeliz Prior, Simone Battista, William J Gregory, Patricia Bisset, Sandra Derham, Dervil M Dockrell, Caroline Livesey, Gemma O’Callaghan

**Affiliations:** School of Health and Society, Centre for Human Movement and Rehabilitation, University of Salford, Greater Manchester, Salford, UK; School of Health and Society, Centre for Human Movement and Rehabilitation, University of Salford, Greater Manchester, Salford, UK; Rheumatology Department, Salford Royal Hospital, Northern Care Alliance NHS Foundation Trust, Greater Manchester, Salford, UK; Faculty of Health and Education, Manchester Metropolitan University, Greater Manchester, Manchester, UK; Rheumatology Department, NHS Greater Glasgow and Clyde, Glasgow, UK; The RNHRD and Brownsword Therapies Centre, Royal United Hospitals Bath NHS Foundation Trust, Bath, UK; Bone Research Group, Rheumatic Diseases, Institute of Genetics and Cancer, Western General Hospital, University of Edinburgh, Edinburgh, UK; Occupational Therapy Service, Department of Rheumatology, Blackpool Teaching Hospitals NHS Foundation Trust, Lancashire, Blackpool, UK; Rehabilitation Department, Freeman Hospital, Newcastle Upon Tyne Hospitals NHS Foundation Trust, Newcastle upon Tyne, UK

**Keywords:** occupational therapy, capabilities framework, professional standards, rheumatology rehabilitation, competency framework

## Abstract

**Objectives:**

An online survey was conducted to explore the clinical roles and expertise of rheumatology occupational therapists (OTs) to inform the development of a UK-specific capabilities framework to enhance care quality and career progression.

**Methods:**

A working group established through the British Society for Rheumatology (BSR) designed and disseminated an online survey via social media and profession-specific networks. Snowball sampling was employed. The survey collected data on job roles, work settings, satisfaction levels, perceived National Health Service Agenda for Change banding appropriateness and comfort with the European Alliance of Associations for Rheumatology (EULAR) Core Competencies. Responses underwent descriptive analysis.

**Results:**

Eighty-eight rheumatology OTs participated in the survey. Most worked full time (18.2%) at Band 6 (44.3%) or Band 7 (46.6%), primarily in acute settings (73.9%). The majority (75–90%) dedicated their time to direct clinical contact, with 75% feeling their job description accurately reflected their role and 23% reporting they had not had sufficient formal training to perform their job role. Participants performed a wide range of job roles, including assessment and advice on activities of daily living (97.7%), hand function (100%), self-management education (96.6%) and fatigue management education (95.5%). Comfort with applying EULAR competency recommendations was generally high, but 11% reported difficulty assessing the educational needs of people with rheumatic and musculoskeletal diseases and 9% with the ability to select and apply outcome measures.

**Conclusion:**

The findings highlight the need for a structured capabilities framework for UK OTs in rheumatology to improve standardisation, career progression and quality of care.

Key messages

- Our study provides insights into the training, role, development and challenges of occupational therapists in their practice.
- A dedicated capabilities framework is essential to standardise and support UK rheumatology occupational therapists.
- A capabilities framework would enhance career progression, workforce retention and patient care quality.

## Introduction

Occupational therapists (OTs) play a vital role in the care of individuals with rheumatic and musculoskeletal diseases (RMDs) [1, 2]. In rheumatology, OTs work as part of a multidisciplinary team, collaborating with rheumatologists, nurses, physiotherapists, podiatrists, pharmacists and psychologists to deliver holistic care. Their role extends beyond functional rehabilitation, encompassing psychological support, upper limb orthotic provision, pain and fatigue management, work rehabilitation and education on joint protection and energy conservation [1, 3]. OTs play a key role in workplace assessments, advising on reasonable adjustments to help individuals with RMDs remain employed or return to work [4, 5]. Additionally, they assess and recommend assistive devices and home adaptations to enhance independent living, aiming to prevent disability and improve long-term health outcomes [6].

While capabilities frameworks exist for other UK health professionals in rheumatology, there is currently no equivalent framework for OTs. The Rheumatology Physiotherapy Capabilities Framework provides a structured approach to screening, assessment and specialist management of rheumatology conditions [7, 8]. Similarly, the Competency Framework for Rheumatology Nurses [9] outlines expected skills and professional development pathways for nurses working in rheumatology. These frameworks standardise practice and support workforce development, providing clear pathways for career progression and service delivery improvement [7, 9]. The absence of a comparable framework for OTs highlights a gap in the professional landscape that must be addressed to ensure equity across disciplines.

Previous studies indicate that structured competency frameworks enhance job satisfaction, improve professional identity and increase workforce retention among allied health professionals [10]. Therefore, this study aimed to conduct a scoping survey, an exploratory, broad-based questionnaire designed to explore the current roles, expertise and professional development needs of UK rheumatology OTs. The findings will inform the development of a tailored capabilities framework to support career progression, standardise practice and ultimately improve patient outcomes.

## Methods

### Study design and setting

This study is a cross-sectional survey conducted among UK OTs in rheumatology. The survey gathered information on UK OTs’ knowledge, skills and capabilities. Ethical approval was granted by the University of Salford’s School of Health and Society Ethics Panel (Reference: 140 on 19 March 2024). All participants provided informed consent digitally by selecting a consent checkbox before completing the survey. The study was conducted according to the principles of the Declaration of Helsinki, ensuring ethical standards were upheld to protect the rights and welfare of participants, and reported following the Strengthening the Reporting of Observational studies in Epidemiology (STROBE) statement [11] and the Checklist for Reporting Results of Internet E-Surveys (CHERRIES) [12] (Supplementary Files S1 and S2, available at *Rheumatology Advances in Practice* online).

### Participants

OTs practising in the UK who considered rheumatology as a significant part of their clinical practice, including those in dedicated roles or treating a substantial rheumatology caseload, were eligible to participate. Participants were identified through professional organisations such as the British Society for Rheumatology (BSR) and the Royal College of Occupational Therapy (RCOT), as well as through professional networks and social media platforms. Participants were recruited via e-mail invitations sent through BSR and RCOT membership channels, professional networks and social media posts. Snowball sampling also encouraged participants to share the survey with eligible colleagues.

### Data collection

A working group consisting of six rheumatology OTs, one rheumatology physiotherapist (previously involved in the development of the physiotherapy capabilities framework) and two researchers was established through the BSR to co-develop the survey. The draft survey was piloted with 17 rheumatology occupational therapists to assess clarity and usability, and minor adjustments to wording and layout were made based on their feedback. The first section (the participation information sheet and informed consent form) provided the participants with detailed information about the study’s purpose, procedures and rights. Before proceeding with the survey, consent was obtained electronically.

The first few questions explored participants’ job role information, including employment status, whether rheumatology occupational therapy constituted their full or partial role and whether they worked full time or part time. National Health Service (NHS) Agenda for Change banding appropriateness was assessed by asking participants to indicate their current job band (ranging from Band 3 to Band 8d/Consultant Occupational Therapist) or to specify an alternative band with a free-text field. At the same time, work settings were identified through multiple-choice options. To capture professional activities, participants reported the percentage of time spent on direct clinical contact *vs* other responsibilities. They rated their job satisfaction across various aspects, such as job description accuracy, time allocation for new and follow-up patients and support from colleagues, using a 5-point Likert scale. The survey also explored specific job roles and responsibilities, with participants indicating whether they performed tasks such as patient assessments, psychological interventions, ergonomic advice, teaching and research involvement. Competency and training were assessed by asking participants to rate their comfort level with the EULAR Core Competencies of Health Professionals in Rheumatology [13] on a 5-point Likert scale, with an option for ‘not applicable’. Additionally, participants evaluated the appropriateness of their job banding in recognising their skill set, selecting responses ranging from ‘very appropriate’ to ‘very inappropriate’. Then participants identified their memberships in relevant organisations, including the RCOT, BSR and EULAR. Finally, an open-ended section allowed participants to provide additional comments or express interest in further involvement in the project. A detailed description of the survey instrument is reported in Supplementary File S3, available at *Rheumatology Advances in Practice* online.

The survey was disseminated online between 18 April and 18 June 2024 through Jisc Online Surveys version 3, a secure web application following strict information security standards (ISO27001) and data processing in compliance with the General Data Protection Regulation. Completion of the survey was anonymous and entirely voluntary. Researchers’ contact details were supplied to enable any questions or concerns to be answered before completing the online survey instrument. Respondents were able to review and change answers before submitting the survey. To submit the survey, all questions had to be answered. Participants could withdraw at any point before submitting their responses and Jisc does not register incomplete surveys. However, since the survey was anonymous, participants could not withdraw their responses after submission. Any optional e-mail addresses provided at the end of the survey for future contact to inform the development of the capabilities framework were stored separately from survey data to maintain confidentiality.

### Data analysis

Survey responses were descriptively analysed using Stata version 18 (StataCorp, College Station, TX, USA). Continuous variables were reported as mean and s.d. Categorical variables were reported as absolute and percentage frequencies. Differences in components of participants’ job roles stratified by band level were descriptively explored.

## Results

A total of 88 OTs participated in the survey. Among them, 18.2% worked full time exclusively in rheumatology, 42.1% worked part time exclusively in rheumatology, 21.6% had rheumatology as part of their full-time job and 18.2% had rheumatology as part of their part-time job (Table 1). The distribution of job bands among participants was as follows: 2.3% were in Band 5, 44.3% in Band 6, 46.6% in Band 7, 4.6% in Band 8a/Advanced Practitioner/Clinical Specialist and 1.1% in Band 8b/c/d or Consultant Occupational Therapist. One participant reported being in another band, i.e. split between Band 8a and Band 7 throughout the week (Table 1). Participants worked in various settings, with the majority (73.9%) based in acute settings or hospital outpatient clinics (rheumatology clinics) (Table 1). Regarding the time spent on direct clinical contact, 5.7% spent <50% of their time on direct clinical contact, 5.7% spent 50%, 15.8% spent 60%, 10.2% spent 70%, 19.3% spent 75%, 21.6% spent 80%, 8.0% spent 85%, 8.0% spent 90% and 5.7% spent >90% (Table 1).

**Table 1.** Descriptive statistics (*N* = 88).

Characteristics	*n* (%)
Job role as rheumatology OT	
100% of job role, full time	16 (18.2)
100% of job role, part time	37 (42.1)
Part of job role, full time	19 (21.6)
Part of job role, part time	16 (18.2)
Band	
5	2 (2.3)
6	39 (44.3)
7	41 (46.6)
8a/advanced practitioner/clinical specialist	4 (4.6)
Band 8b, c or d/consultant OT	1 (1.1)
Other^a^	1 (1.1)
Settings, n (%)^b^	
Acute setting/hospital outpatients (rheumatology clinic)	65 (73.9)
Acute setting/hospital in-patients (rheumatology/mixed ward)	5 (5.7)
Occupational therapy department (acute)	17 (19.3)
Occupational therapy department (community)	9 (10.2)
Primary care/community clinic/GP practice (OT role)	2 (2.8)
Primary care/GP practice (first-contact practitioner)	0 (0.0)
Intermediate care triage service (iCATS, MCATS etc.)	1 (1.1)
Co-located setting with rheumatology department	6 (6.8)
Co-located setting with GP support access or rheumatology GPwSI	0 (0.0)
Other^c^	6 (6.8)
Time spent on direct clinical contact and other activities	
<50% direct clinical contact	5 (5.7)
50% direct clinical contact, 50% other activities	5 (5.7)
60% direct clinical contact, 40% other activities	14 (15.8)
70% direct clinical contact, 30% other activities	9 (10.2)
75% direct clinical contact, 25% other activities	17 (19.3)
80% direct clinical contact, 20% other activities	19 (21.6)
85% direct clinical contact, 15% other activities	7 (8.0)
90% direct clinical contact, 10% other activities	7 (8.0)
>90% direct clinical contact	5 (5.7)
Association, n (%)^b^	
RCOT	73 (83.0)
RCOT Rheumatology Clinical Forum	23 (26.1)
BSR	37 (42.0)
SSR	7 (8.0)
BAHT	15 (17.0)
Vocational Rehabilitation Association UK Network	0 (0.0)
EULAR Health Professionals in Rheumatology	7 (8.0)
WFOT	1 (1.1)
Other^d^	5 (5.7)
None	7 (8.0)

GP: general practitioner; iCATS: Intermediate Care Assessment and Treatment Services; MCATS: Musculoskeletal Clinical Assessment and Triage Services; GPwSI: general practitioner with special interest.

a Split between Band 8a and Band 7 throughout the week.

b Multiple answers available.

c Participants were working in diverse settings, including acute settings within the physiotherapy department, conducting home visits, physiotherapy outpatient departments, paediatric rheumatology, outpatient occupational therapy teams within acute hospitals and outpatient therapy departments in acute hospital settings.

d These include previous subscriptions that were not renewed to some of the above or registration in associations like the National Rheumatoid Arthritis Society and the North West Rheumatology Special Interest Group for Occupational Therapists.

### Professional memberships

Most participants were members of the RCOT (83.0%). Other memberships included the RCOT Rheumatology Clinical Forum (26.1%), BSR (42.0%), Scottish Society for Rheumatology (SSR) (8.0%), British Association of Hand Therapists (BAHT) (17.0%), EULAR Health Professionals in Rheumatology (8.0%) and World Federation of Occupational Therapists (WFOT) (1.1%) (Table 1).

### Job satisfaction

**Figure 1.**
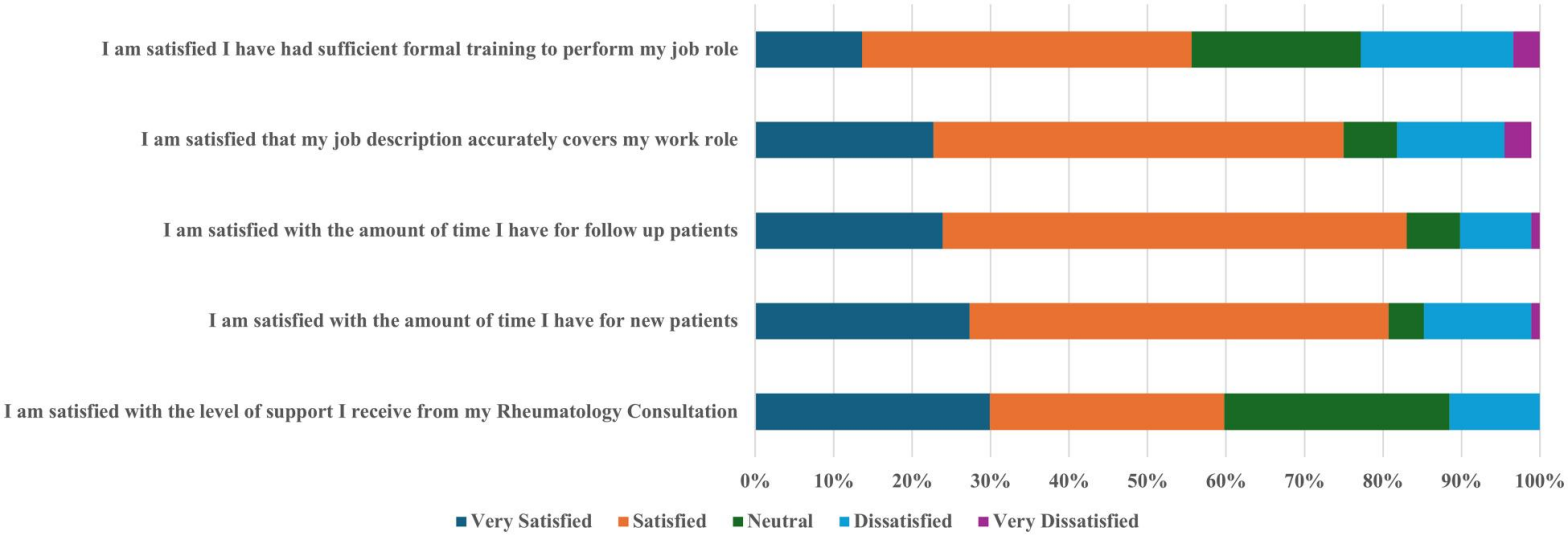
Levels of satisfaction with the job role

Participants reported varying levels of satisfaction with different aspects of their job roles. The majority (75%) were satisfied with their job descriptions and the time available for new (53.4%) and follow-up patients (59.1%). However, satisfaction with the level of support from rheumatology consultants was mixed, with 59.8% either satisfied or very satisfied, 28.7% neutral and 11.5% dissatisfied. Formal training adequacy was an area that needed attention, with 42% satisfied and 13.6% very satisfied and 22% being either dissatisfied or very dissatisfied (Fig. 1; Supplementary File S4, available at *Rheumatology Advances in Practice* online).

### Job roles

Participants performed a wide range of job roles, including assessment and advice on activities of daily living (97.7%), hand function (100%), self-management education (96.6%) and fatigue management education (95.5%). Other roles included psychological assessments (33.0%), psychological interventions (48.9%), provision of orthotics (100% off the shelf, 87.5% custom made) and ergonomic advice (90.9%). See Table 2 for the complete list. Supplementary File S5, available at *Rheumatology Advances in Practice* online provides a detailed breakdown of the components of participants’ job roles stratified by band level. For example, assessment and advice on activities of daily living and hand function were consistently performed across all bands. In contrast, more specialised tasks such as psychological assessments and interventions, ergonomic advice and teaching responsibilities were more prevalent among higher band levels.

**Table 2.** Components of participants’ job role (*N* = 88)

Job role	Yes, *n* (%)	No, *n* (%)
Assessment and advice on activities of daily living (e.g. self-care, productivity and leisure)	86 (97.7)	2 (2.3)
Assessment and advice on hand function	88 (100.0)	0 (0.0)
Assessing educational needs and psychological status	56 (63.6)	32 (36.4)
Self-management education	85 (96.6)	3 (3.4)
Running self-management group education program (e.g. fatigue, joint protection)	37 (42.0)	51 (58.0)
Sexual health and sexual dysfunction education	18 (20.5)	70 (79.5)
Psychological assessment	29 (33.0)	59 (67.0)
Psychological interventions	43 (48.9)	45 (51.1)
Fatigue management education	84 (95.5)	4 (4.5)
Sleep assessment and education^a^	58 (65.9)	30 (34.1)
Insomnia assessment	2 (3.4)	56 (96.6)
Sleep apnoea assessment	5 (8.6)	53 (91.4)
Sleep hygiene	58 (100.0)	0 (0.0)
Hand exercises to improve/maintain range of movement, muscle strength and endurance	88 (100.0)	0 (0.0)
Pain management	75 (85.2)	13 (14.8)
Mood management	48 (54.5)	40 (45.5)
Provision of wrist and hand orthotics (off the shelf)	88 (100.0)	0 (0.0)
Provision of custom-made wrist and hand orthotics	77 (87.5)	11 (12.5)
Provision of compression/arthritis gloves	64 (72.7)	24 (27.3)
Ergonomic approaches to reduce pain, fatigue and joint strain	80 (90.9)	8 (9.1)
Using ergonomic equipment and assistive technology	60 (68.2)	28 (31.8)
Work advice (e.g. brief advice on job retention/return to work)	74 (84.1)	14 (15.9)
Job retention vocational/work rehabilitation intervention	32 (36.4)	56 (63.6)
Return-to-work vocational/work rehabilitation intervention	33 (37.5)	55 (62.5)
Health promotion	63 (71.6)	25 (28.4)
Tai chi for arthritis	1 (1.1)	87 (98.9)
Home ADL assessment (i.e. for people with chronic physical functional problems)	39 (44.3)	49 (55.7)
Environmental assessment (i.e. assessing the patient’s home)	35 (39.8)	53 (60.2)
Workplace visits	11 (12.5)	77 (87.5)
Regional or national expertise in occupational therapy for rare diagnoses	7 (8.0)	81 (92.0)
csDMARD monitoring	1 (1.1)	87 (98.9)
Biologics (and JAK inhibitor) monitoring	0 (0.0)	88 (100.0)
Input into databases, e.g. BlueTeq	1 (1.1)	87 (98.9)
Injection therapy	6 (6.8)	82 (93.2)
Ultrasound scanning	2 (2.3)	86 (97.7)
Non-medical prescribing	6 (6.8)	82 (93.2)
Triaging incoming rheumatology referrals	45 (51.1)	43 (48.9)
New patient clinic for rheumatology referrals	22 (25.0)	66 (75.0)
Bath scoring for SpA/axial SpA	3 (3.4)	85 (96.6)
28-joint DAS RA counts	10 (11.4)	78 (88.6)
PsARC joint counts	2 (2.3)	86 (97.7)
Performing annual reviews assessments (e.g. cardiac, bone health etc.)	2 (2.3)	86 (97.7)
MRI scan requests	3 (3.4)	85 (96.6)
X-ray requests	7 (8.0)	81 (92.0)
DEXA scan requests	1 (1.1)	87 (98.9)
Ultrasound requests	7 (8.0)	81 (92.0)
Requesting blood tests	4 (4.5)	84 (95.5)
Requesting nerve conduction tests	8 (9.1)	80 (90.9)
Referral to (other) AHP services	67 (76.1)	21 (23.9)
Referral to clinical health psychology/IAPT	36 (40.9)	52 (59.1)
Referral to pain clinic	36 (40.9)	52 (59.1)
Autonomous/direct referral to orthopaedics	15 (17.0)	73 (83.0)
Letters of support (e.g. housing, benefits, education)	71 (80.7)	17 (19.3)
Teaching of medical students/trainees observing your clinics	45 (51.1)	43 (48.9)
Teaching of AHPs/nurses (and AHP and nursing students) observing your clinics	68 (77.3)	20 (22.7)
Formal teaching for medical staff/students/AHPs/nurses	30 (34.1)	58 (65.9)
Supervision of less experienced rheumatology colleagues	55 (62.5)	33 (37.5)
Formal teaching of occupational therapists	33 (37.5)	55 (62.5)
Lecturing for higher education institutions	8 (9.1)	80 (90.9)
Contribution to research (e.g. data collection, recruitment, intervention delivery)	40 (45.5)	48 (54.5)
Leading of research projects and audits	24 (27.3)	64 (72.7)
Other^b^	7 (8.0)	81 (92.0)

ADL: activities of daily living; csDMARD: conventional synthetic DMARD; JAK: Janus kinase; PsARC: Psoriatic Arthritis Response Criteria; AHP: allied healthcare professional; IAPT: improving access to psychological therapies.

a *n* = 58 (those who answered yes to ‘sleep assessment and education’).

b Other activities such as peer mentoring, tailored exercises, supervision of junior physiotherapists, service development and workshop creation.

### Band appropriateness

Regarding the appropriateness of their job banding, 26 (29.6%) felt their band was very appropriate, 34 (38.6%) mostly appropriate, 14 (15.9%) were unsure, 10 (11.3%) felt it was mostly inappropriate and 4 (4.6%) felt it was very inappropriate.

### Comfort with EULAR core competencies

**Figure 2.**
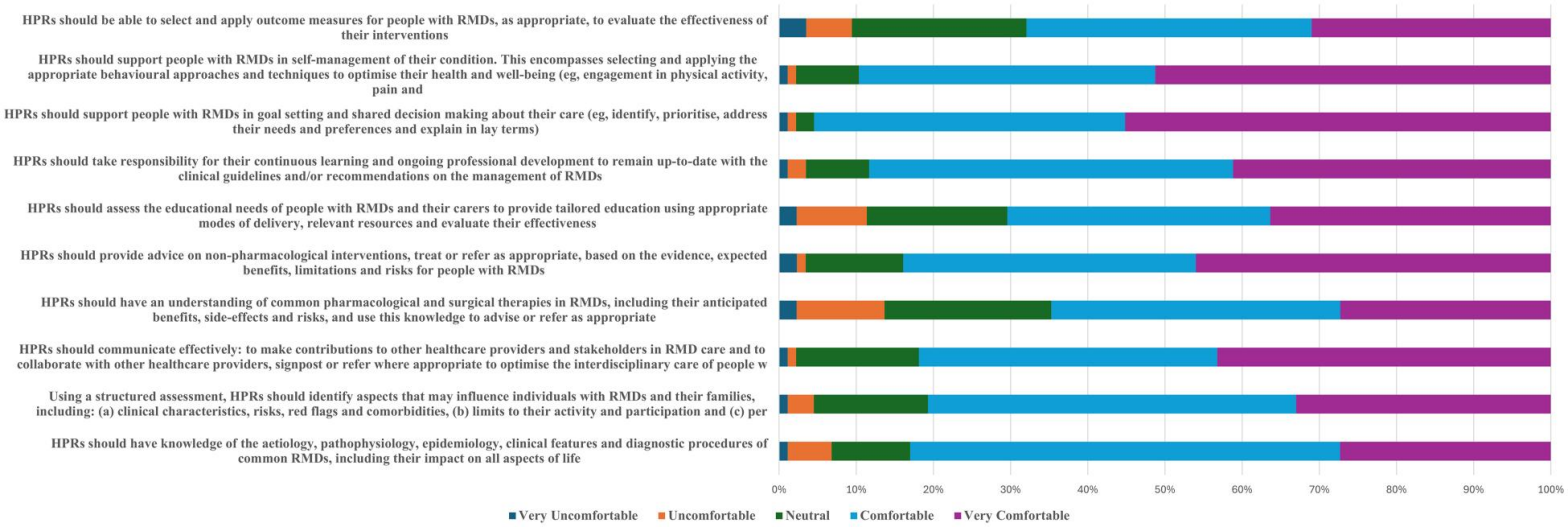
Self-assessment of comfort level with EULAR core competencies in day-to-day practice

Participants’ self-assessment of comfort with EULAR core competencies varied. The majority felt comfortable or very comfortable with knowledge of RMDs (55.7% comfortable, 27.4% very comfortable), structured assessments (47.7% comfortable, 33.0% very comfortable) and effective communication (38.6% comfortable, 43.2% very comfortable). However, there were areas where participants felt less comfortable, such as pharmacological and surgical therapies (37.5% comfortable, 27.3% very comfortable), the use of outcome measures (35.6% comfortable, 29.9% very comfortable) and the importance of assessing the educational needs of patients and caregivers to tailor the intervention (34.1% comfortable, 36.4% very comfortable) (Fig. 2, Supplementary File S6, available at *Rheumatology Advances in Practice* online).

## Discussion

The findings of this study highlight the diverse and evolving roles of rheumatology OTs across different healthcare settings in the UK. There is no central record of rheumatology OTs in the UK. However, the most recent national estimate indicates 167 rheumatology departments across the UK [14]. BSR workforce data show that only 52% of services have an OT embedded in the multidisciplinary team, suggesting ≈87 departments currently include an OT [15]. Our 88 survey responses are therefore likely to represent a substantial proportion of the rheumatology OT workforce. This is comparable to the national physiotherapy workforce survey, which received 97 responses [8].

These results align with previous research indicating that rheumatology OTs are central to rheumatology multidisciplinary teams, primarily delivering direct patient care [13, 14]. Nevertheless, not all rheumatology departments have OTs working in multidisciplinary teams. This survey showed the key role OTs play in multidisciplinary teams and suggests potential value in ensuring the presence Ots across departments. Unlike rheumatology physiotherapists and nurses, who have structured competency frameworks guiding their professional development, OTs lack a standardised framework, leading to variations in role expectations, access to training and professional recognition, impacting the quality of patient care.

A key finding of this study was that 23% of respondents felt they had not received sufficient formal training to perform their job roles effectively. Rather, they have reached their current level of capability by informal and on-the-job training and experience. This highlights the need for a structured training pathway to ensure consistency in clinical competencies. The Rheumatology Physiotherapy Capabilities Framework provides a structured approach to developing and assessing specialist physiotherapy skills, aligning them with professional development pathways and NHS workforce planning [10]. The absence of a comparable framework for OTs suggests a professional disparity that could be addressed through the development of a standardised capabilities framework.

Another significant issue identified in the study was the variation in job banding appropriateness. These discrepancies suggest inconsistencies in job evaluation processes and professional recognition within rheumatology occupational therapy. The capability and competency frameworks for rheumatology physiotherapists and nurses provide clear benchmarks for career progression, ensuring that skills and responsibilities align with job banding structures. Implementing a similar framework for rheumatology OTs would enhance transparency in job evaluation, support workforce development and ensure equity across disciplines.

Additionally, the survey explored participants’ self-assessment of comfort with EULAR core competencies [13], and most respondents reported confidence in core assessments, patient education and multidisciplinary communication. This aligns with previous research highlighting gaps in professional training for non-medical health professionals in pharmacology and patient education [16]. A dedicated capabilities framework for rheumatology OTs could incorporate structured training modules to address these gaps, ensuring practitioners have the necessary competencies to deliver holistic patient care.

This study has several limitations. The use of snowball sampling could have introduced selection bias, favouring those more engaged in professional networks or with stronger views on the topic. Additionally, the absence of a validated measure of clinical competence led us to assess perceived comfort with tasks rather than objective capability, which may limit the interpretation of skill levels. Finally, as the survey was exploratory in nature, findings should be interpreted as indicative rather than definitive, guiding the next steps in framework development.

This study provides valuable insights into the roles, training needs and professional development challenges rheumatology OTs face in the UK. Future research should focus on co-developing and implementing this framework, ensuring that it reflects the evolving needs of rheumatology OTs and aligns with best practices in clinical excellence and workforce sustainability.

## Supplementary Material

rkaf072_Supplementary_Data

## Data Availability

The data underlying this article will be shared upon reasonable request to the corresponding author.
