# Supplementary material for: A scoping survey for the UK rheumatology occupational therapy capabilities framework
Source: Rheumatol Adv Pract. 2025 Jun 9;9(3):rkaf072. doi: 10.1093/rap/rkaf072 (PMC12202759; doi:10.1093/rap/rkaf072)
Supplement: rkaf072_Supplementary_Data [file rkaf072_supplementary_data.zip › 25-064 Supplementary Data S3.docx]

**Supplementary Data S3 - UK-Wide Rheumatology Occupational Therapy Specialist Professional Knowledge, Skills & Capability Framework Survey**

This survey aims to support the development of a specialist Rheumatology professional framework that covers levels, roles and scopes of occupational therapy practice across UK settings.

Thank you for taking part. Your participation will help provide valuable information towards this project.

1. Do you currently work (for all or part of your job) as a Rheumatology Occupational Therapist?
   1. Yes, Rheumatology Occupational Therapy is 100% of my job role, I work full-time.
   2. Yes, Rheumatology Occupational Therapy is 100% of my job role, I work part-time.
   3. Yes, Rheumatology Occupational Therapy is a part of my job role, I work full-time.
   4. Yes, Rheumatology Occupational Therapy is a part of my job role, I work part-time.
   5. No, I do not currently work in Rheumatology Occupational Therapy.
2. What level are you currently employed at for your Rheumatology Occupational Therapy role?
   1. Band 3
   2. Band 4
   3. Band 5
   4. Band 6
   5. Band 7
   6. Band 8a/ Advanced Practitioner / Clinical Specialist
   7. Band 8b or c or d / Consultant Occupational Therapist
   8. Other (please specify):

Free text box: ------------------------------------

1. Where are you based when you see your Rheumatology caseload? (tick all that apply)
   1. Acute setting / Hospital outpatients (Rheumatology Clinic)
   2. Acute setting / Hospital in-patients (Rheumatology / Mixed ward)
   3. Physiotherapy department (Acute)
   4. Physiotherapy department (Community)
   5. Primary care /Community Clinic / GP practice (OT role)
   6. Primary care / GP practice (first contact practitioner)
   7. Intermediate care triage service (iCATS, MCATS, etc.)
   8. Co-located setting with Rheumatology Department
   9. Co-located setting with GP support access or Rheum GPwSI
   10. Other (please specify):
   11. Free text box: ------------------------------------
2. Thinking about the Rheumatology part of your job role, what percentage of your time is spent undertaking Direct Clinical Contact or other activities?
   - 1. less than 50% direct clinical contact
     2. 50% direct clinical contact; 50% other activities
     3. 60% direct clinical contact; 40% other activities
     4. 70% direct clinical contact; 30% other activities
     5. 75% direct clinical contact; 25% other activities
     6. 80% direct clinical contact; 20% other activities
     7. 85% direct clinical contact; 15% other activities
     8. 90% direct clinical contact; 10% other activities
     9. more than 90% direct clinical contact

Comments: ----------------------------------

1. Please rate the following statements covering your work role.

|  | Very Dissatisfied | Dissatisfied | Neutral | Satisfied | Very Satisfied | N/A |
| --- | --- | --- | --- | --- | --- | --- |
| I am satisfied that my job description accurately covers my work role |  |  |  |  |  |  |
| I am satisfied with the amount of time I have for new patients |  |  |  |  |  |  |
| I am satisfied with the amount of time I have for follow up patients |  |  |  |  |  |  |
| I am satisfied with the level of support I receive from my Rheumatology Consultant colleagues |  |  |  |  |  |  |
| I am satisfied I have had sufficient formal training to perform my job role |  |  |  |  |  |  |

Comments: ----------------------------------

1. My Rheumatology job role includes...( tick all boxes that apply)
   1. Assessment & advice on activities of daily living (e.g., self-care, productivity and leisure)
   2. Assessment & advice on hand function
   3. Assessing educational needs and psychological status
   4. Self-management education
   5. Running self-management group education programme (e.g., fatigue, joint protection, hand exercises)
   6. Sexual health and sexual dysfunction education
   7. Psychological assessment
   8. Psychological interventions (e.g., CBT approaches to pain management, Acceptance and Commitment Therapy (ACT), motivational interviewing techniques, self-esteem and self-efficacy building, coping skills).
   9. Fatigue management education
   10. Insomnia
   11. Sleep assessment and education – if yes:
   12. Insomnia assessment

11.2 Sleep apnoea assessment

11.3 Sleep hygiene

- 1. Hand exercises to improve/ maintain range of movement, muscle strength and endurance.
  2. Pain management
  3. Mood management
  4. Provision of wrist and hand orthotics (off the shelf)
  5. Provision of custom-made wrist and hand orthotics
  6. Provision of compression/ arthritis gloves
  7. Ergonomic approaches to reduce pain, fatigue and joint strain.
  8. Using ergonomic equipment and assistive technology
  9. Work advice (e.g. brief advice on job retention/ return-to-work)
  10. Job retention vocational/ work rehabilitation intervention
  11. Return-to-work vocational/ work rehabilitation intervention
  12. Health promotion
  13. Tai Chi for Arthritis
  14. Home ADL assessment (i.e., for people with chronic physical functional problems or psychological problems affecting function at home need to be assessed at home)
  15. Environmental assessment (i.e., assessing the patient’s home and to see what equipment they have in situ and the environment-space, size, doors, steps and stairs and identify any equipment or adaptations that may be needed for discharge)
  16. Workplace visits
  17. Regional or National expertise in occupational therapy for rare diagnoses
  18. csDMARD monitoring
  19. Biologics (and JAK-inhibitor) monitoring
  20. Input onto databases, eg. BlueTeq
  21. Injection therapy
  22. Ultrasound scanning
  23. Non-Medical Prescribing
  24. Triaging in-coming Rheumatology Referrals
  25. New Patient clinic for Rheumatology Referrals
  26. Bath scoring for Spondyloarthritis / Axial Spondyloarthritis
  27. DAS-28 Rheumatoid Arthritis Joint Counts
  28. PsARC Joint Counts
  29. Performing annual reviews assessments (eg. cardiac, bone health, etc.)
  30. MR scan requests
  31. XR requests
  32. DEXA scan requests
  33. Ultrasound requests
  34. Requesting blood tests
  35. Requesting nerve conduction tests
  36. Referral to (other) AHP services
  37. Referral to Clinical Health Psychology / IAPT
  38. Referral to Pain Clinic
  39. Autonomous / direct referral to orthopaedics
  40. Letters of support (e.g., housing, benefits, education)
  41. Teaching of medical students / trainees observing your clinics
  42. Teaching of AHPs / nurses (& AHP and nursing students) observing your clinics
  43. Formal teaching for medical staff / students / AHPs / nurses
  44. Supervision of less experienced rheumatology colleagues
  45. Formal teaching of occupational therapists
  46. Lecturing for Higher Education Institutions
  47. Contribution to research (e.g., data collection, recruitment, intervention delivery)
  48. Leading of research projects and audits
  49. Other (please specify):-------------------------------

1. Do you feel that your job banding appropriately recognizes your skillset?
   1. Yes, my band is very appropriate for the job roles I perform.
   2. Yes, my band is mostly appropriate for the job roles I perform.
   3. I am unsure if my banding appropriately reflects the job roles I perform.
   4. No, my band is mostly inappropriate for the job roles I perform.
   5. No, my band is very inappropriate for the job roles I perform.
   6. Other (please specify): ------------------------------
2. Please rate how comfortable you feel in your day-to-day practice in relation to these EULAR recommendations for core competencies (HPR = Health Professional in Rheumatology; RMD = Rheumatic and Musculoskeletal Diseases)

|  | Very Uncomfortable | Uncomfortable | Neutral | Comfortable | Very Comfortable | N/A |
| --- | --- | --- | --- | --- | --- | --- |
| HPRs should have knowledge of the aetiology, pathophysiology, epidemiology, clinical features and diagnostic procedures of common RMDs, including their impact on all aspects of life |  |  |  |  |  |  |
| Using a structured assessment, HPRs should identify aspects that may influence individuals with RMDs and their families, including: (a) clinical characteristics, risks, red flags and comorbidities, (b) limits to their activity and participation and (c) personal and environmental factors |  |  |  |  |  |  |
| HPRs should communicate effectively: to make contributions to other healthcare providers and stakeholders in RMD care and to collaborate with other healthcare providers, signpost or refer where appropriate to optimise the interdisciplinary care of people with RMDs |  |  |  |  |  |  |
| HPRs should have an understanding of common pharmacological and surgical therapies in RMDs, including their anticipated benefits, side-effects and risks, and use this knowledge to advise or refer as appropriate |  |  |  |  |  |  |
| HPRs should provide advice on non-pharmacological interventions, treat or refer as appropriate, based on the evidence, expected benefits, limitations and risks for people with RMDs |  |  |  |  |  |  |
| HPRs should assess the educational needs of people with RMDs and their carers to provide tailored education using appropriate modes of delivery, relevant resources and evaluate their effectiveness |  |  |  |  |  |  |
| HPRs should take responsibility for their continuous learning and ongoing professional development to remain up-to-date with the clinical guidelines and/or recommendations on the management of RMDs |  |  |  |  |  |  |
| HPRs should support people with RMDs in goal setting and shared decision making about their care (eg, identify, prioritise, address their needs and preferences and explain in lay terms) |  |  |  |  |  |  |
| HPRs should support people with RMDs in self-management of their condition. This encompasses selecting and applying the appropriate behavioural approaches and techniques to optimise their health and well-being (eg, engagement in physical activity, pain and fatigue management) |  |  |  |  |  |  |
| HPRs should be able to select and apply outcome measures for people with RMDs, as appropriate, to evaluate the effectiveness of their interventions |  |  |  |  |  |  |

1. Are you a member (or follower) of any of the following?
   1. RCOT (Royal College of Occupational Therapists)
   2. RCOT Rheumatology Clinical Forum
   3. BSR (British Society for Rheumatology)
   4. SSR (Scottish Society for Rheumatology)
   5. BAHT (British Association of Hand Therapists)
   6. VRA (Vocational Rehabilitation Association UK) Network
   7. EULAR (HPR) Health Professionals in Rheumatology
   8. WFOT (World Federation of Occupational Therapists) Occupational Therapy International Online Network (OTION)
   9. Other (Please specify): ------------------
2. Thank you so much for your time in filling in this survey.

Please provide any comments or suggestions you have that relate to this project to collate professional knowledge, skills & capability framework for rheumatology occupational therapy.

Also, if you are interested in being contacted about further development work on this framework, please provide an email contact. (This information will not be shared or used for any other purpose)

Email:…………………………………… (Optional)
