# Supplementary material for: A scoping survey for the UK rheumatology occupational therapy capabilities framework
Source: Rheumatol Adv Pract. 2025 Jun 9;9(3):rkaf072. doi: 10.1093/rap/rkaf072 (PMC12202759; doi:10.1093/rap/rkaf072)
Supplement: rkaf072_Supplementary_Data [file rkaf072_supplementary_data.zip › 25-064 Supplementary Data S4.docx]

**Supplementary Data S4 - Levels of Satisfaction with the Job Role**

|  | n=88 (100) | | | | | |
| --- | --- | --- | --- | --- | --- | --- |
| **Statements, n (%)** | **Very Dissatisfied** | **Dissatisfied** | **Neutral** | **Satisfied** | **Very Satisfied** | **NA** |
| I am satisfied that my job description accurately covers my work role | 3 (3.4) | 12 (13.6) | 6 (6.8) | 46 (52.3) | 20 (22.7) | 1 (1.1) |
| I am satisfied with the amount of time I have for new patients | 1 (1.1) | 12 (13.6) | 4 (4.5) | 47 (53.4) | 24 (27.3) | 0 (0.0) |
| I am satisfied with the amount of time I have for follow up patients | 1 (1.1) | 8 (9.1) | 6 (6.8) | 52 (59.1) | 21 (23.9) | 0 (0.0) |
| I am satisfied with the level of support I receive from my Rheumatology Consultation | 0 (0) | 10 (11.5) | 25 (28.7) | 26 (29.9) | 26 (29.9) | 0 (0.0) |
| I am satisfied I have had sufficient formal training to perform my job role | 3 (3.4) | 17 (19.3) | 19 (21.6) | 37 (42) | 12 (13.6) | 0 (0.0) |

Legend: n, number; NA, not applicable.
