# Supplementary material for: A scoping survey for the UK rheumatology occupational therapy capabilities framework
Source: Rheumatol Adv Pract. 2025 Jun 9;9(3):rkaf072. doi: 10.1093/rap/rkaf072 (PMC12202759; doi:10.1093/rap/rkaf072)
Supplement: rkaf072_Supplementary_Data [file rkaf072_supplementary_data.zip › 25-064 Supplementary Data S5.docx]

**Supplementary Data S5- Component of Participants’ Job Role Stratified By Band Level**

| **Job Role** | **Band 5 (n=2)** | **Band 6**  **(n=39)** | **Band 7**  **(n=41)** | **Band 8a/ Advanced Practitioner / Clinical Specialist (n=4)** | **Band 8b or c or d / Consultant Occupational Therapist (n=1)** | **Other (n=1)** |
| --- | --- | --- | --- | --- | --- | --- |
| Assessment & advice on activities of daily living (e.g., self-care, productivity and leisure) (n=86) | 2 (2.3) | 39 (45.4) | 40 (46.5) | 3 (3.5) | 1 (1.2) | 1 (1.2) |
| Assessment & advice on hand function (n=88) | 2 (2.3) | 39 (44.3) | 41 (46.6) | 4 (4.6) | 1 (1.1) | 1 (1.1) |
| Assessing educational needs and psychological status (n=56) | 0 (0.0) | 26 (46.4) | 27 (48.2) | 2 (3.6) | 0 (0.0) | 1 (1.8) |
| Self-management education (n=85) | 2 (2.4) | 38 (44.7) | 40 (47.1) | 4 (4.7) | 0 (0.0) | 1 (1.2) |
| Running self-management group education programme (e.g., fatigue, joint protection) (n=37) | 0 (0.0) | 15 (40.5) | 20 (54.1) | 1 (2.7) | 1 (2.7) | 0 (0.0) |
| Sexual health and sexual dysfunction education (n=18) | 0 (0.0) | 7 (38.9) | 9 (50.0) | 1 (5.6) | 1 (5.6) | 0 (0.0) |
| Psychological assessment (n=29) | 0 (0.0) | 9 (31.0) | 17 (58.5) | 1 (3.5) | 1 (3.5) | 1 (3.5) |
| Psychological interventions (n=43) | 0 (0.0) | 18 (41.8) | 22 (51.2) | 2 (4.7) | 1 (2.3) | 0 (0.0) |
| Fatigue management education (n=84) | 2 (2.4) | 38 (45.2) | 40 (47.6) | 2 (2.4) | 1 (1.2) | 1 (1.2) |
| Sleep assessment and education (n=58) | 1 (1.7) | 24 (41.4) | 29 (50.0) | 2 (3.5) | 1 (1.7) | 1 (1.7) |
| Hand exercises to improve/ maintain range of movement, muscle strength and endurance (n=88) | 2 (2.3) | 39 (44.3) | 41 (46.6) | 4 (4.6) | 1 (1.1) | 1 (1.1) |
| Pain management (n=75) | 0 (0.0) | 33 (44.0) | 38 (50.7) | 2 (2.7) | 1 (1.3) | 1 (1.3) |
| Mood management (n=48) | 0 (0.0) | 23 (47.9) | 23 (47.9) | 1 (2.1) | 1 (2.1) | 0 (0.0) |
| Provision of wrist and hand orthotics (off the shelf) (n=88) | 2 (2.3) | 39 (44.3) | 41 (46.6) | 4 (4.7) | 1 (1.1) | 1 (1.1) |
| Provision of custom-made wrist and hand orthotics (n=77) | 2 (2.6) | 35 (45.5) | 35 (45.5) | 4 (5.2) | 0 (0.0) | 1 (1.3) |
| Provision of compression/ arthritis gloves (n=64) | 2 (3.1) | 27 (42.2) | 32 (50.0) | 2 (3.1) | 1 (1.6) | 0 (0.0) |
| Ergonomic approaches to reduce pain, fatigue and joint strain (n=80) | 2 (2.5) | 36 (45.0) | 38 (47.5) | 3 (3.8) | 1 (1.3) | 0 (0.0) |
| Using ergonomic equipment and assistive technology (n=60) | 2 (3.3) | 25 (41.7) | 30 (50.0) | 2 (3.3) | 1 (1.7) | 0 (0.0) |
| Work advice (e.g. brief advice on job retention/ return-to-work) (n=74) | 2 (2.7) | 33 (44.6) | 35 (47.3) | 3 (4.1) | 1 (1.4) | 0 (0.0) |
| Job retention vocational/ work rehabilitation intervention (n=32) | 0 (0.0) | 15 (46.9) | 15 (46.9) | 1 (3.1) | 1 (3.1) | 0 (0.0) |
| Return-to-work vocational/ work rehabilitation intervention (n=33) | 0 (0.0) | 14 (42.5) | 17 (51.5) | 1 (3.0) | 1 (3.0) | 0 (0.0) |
| Health promotion (n=63) | 0 (0.0) | 29 (46.0) | 30 (47.6) | 2 (3.2) | 1 (1.6) | 1 (1.6) |
| Tai Chi for Arthritis (n=1) | 0 (0.0) | 1 (100) | 0 (0.0) | 0 (0.0) | 0 (0.0) | 0 (0.0) |
| Home ADL assessment (i.e., for people with chronic physical functional problems) (n=39) | 1 (2.6) | 18 (46.2) | 19 (48.7) | 0 (0.0) | 0 (0.0) | 1 (2.5) |
| Environmental assessment (i.e., assessing the patient's home) (n=35) | 1 (2.9) | 16 (45.7) | 16 (45.7) | 0 (0.0) | 1 (2.9) | 1 (2.9) |
| Workplace visits (n=11) | 0 (0.0) | 3 (27.3) | 7 (63.6) | 0 (0.0) | 1 (9.1) | 0 (0.0) |
| Regional or National expertise in occupational therapy for rare diagnoses (n=7) | 0 (0.0) | 1 (14.3) | 3 (42.9) | 3 (42.9) | 0 (0.0) | 0 (0.0) |
| csDMARD monitoring (n=1) | 0 (0.0) | 0 (0.0) | 0 (0.0) | 1 (100.0) | 0 (0.0) | 0 (0.0) |
| Biologics (and JAK-inhibitor) monitoring (n=0) | 0 (0.0) | 0 (0.0) | 0 (0.0) | 0 (0.0) | 0 (0.0) | 0 (0.0) |
| Input onto databases, eg. BlueTeq (n=1) | 0 (0.0) | 0 (0.0) | 1 (100.0) | 0 (0.0) | 0 (0.0) | 0 (0.0) |
| Injection therapy (n=6) | 0 (0.0) | 0 (0.0) | 3 (50.0) | 3 (50.0) | 0 (0.0) | 0 (0.0) |
| Ultrasound scanning (n=2) | 0 (0.0) | 0 (0.0) | 2 (100.0) | 0 (0.0) | 0 (0.0) | 0 (0.0) |
| Non-Medical Prescribing (n=6) | 0 (0.0) | 2 (33.3) | 4 (66.7) | 0 (0.0) | 0 (0.0) | 0 (0.0) |
| Triaging in-coming Rheumatology Referrals (n=45) | 0 (0.0) | 19 (42.2) | 22 (49.0) | 2 (4.44) | 1 (2.2) | 1 (2.2) |
| New Patient clinic for Rheumatology Referrals (n=22) | 0 (0.0) | 8 (36.4) | 12 (54.6) | 1 (4.6) | 1 (4.6) | 0 (0.0) |
| Bath scoring for Spondyloarthritis / Axial Spondyloarthritis (n=3) | 0 (0.0) | 0 (0.0() | 2 (66.7) | 0 (0.0) | 1 (33.3) | 0 (0.0) |
| DAS-28 Rheumatoid Arthritis Joint Counts (n=10) | 0 (0.0) | 1 (10.0) | 6 (60.0) | 3 (30.0) | 0 (0.0) | 0 (0.0) |
| PsARC Joint Counts (n=2) | 0 (0.0) | 0 (0.0) | 1 (50.0) | 1 (50.0) | 0 (0.0) | 0 (0.0) |
| Performing annual reviews assessments (e.g. cardiac, bone health, etc.) (n=2) | 0 (0.0) | 0 (0.0) | 2 (100.0) | 0 (0.0) | 0 (0.0) | 0 (0.0) |
| MR scan requests (n=3) | 0 (0.0) | 0 (0.0) | 1 (33.3) | 2 (66.7) | 0 (0.0) | 0 (0.0) |
| XR requests (n=7) | 0 (0.0) | 0 (0.0) | 5 (71.4) | 2 (28.6) | 0 (0.0) | 0 (0.0) |
| DEXA scan requests (n=1) | 0 (0.0) | 0 (0.0) | 1 (100.0) | 0 (0.0) | 0 (0.0) | 0 (0.0) |
| Ultrasound requests (n=7) | 0 (0.0) | 1 (14.3) | 3 (42.9) | 3 (42.9) | 0 (0.0) | 0 (0.0) |
| Requesting blood tests (n=4) | 0 (0.0) | 0 (0.0) | 1 (25.0) | 3 (74.0) | 0 (0.0) | 0 (0.0) |
| Requesting nerve conduction tests (n=8) | 0 (0.0) | 0 (0.0) | 5 (62.5) | 3 (37.5) | 0 (0.0) | 0 (0.0) |
| Referral to (other) AHP services (n=67) | 1 (1.5) | 27 (40.3) | 34 (50.8) | 4 (6.0) | 1 (1.4) | 0 (0.0) |
| Referral to Clinical Health Psychology / IAPT (n=36) | 1 (2.8) | 11 (30.6) | 20 (55.6) | 2 (5.6) | 1 (2.8) | 1 (2.8) |
| Referral to Pain Clinic (n=36) | 0 (0.0) | 9 (25.0) | 23 (63.9) | 3 (8.3) | 1 (2.8) | 0 (0.0) |
| Autonomous / direct referral to orthopaedics (n=15) | 0 (0.0) | 4 (26.7) | 8 (53.3) | 3 (20.0) | 0 (0.0) | 0 (0.0) |
| Letters of support (e.g., housing, benefits, education) (n=71) | 1 (1.4) | 30 (42.3) | 35 (49.3) | 3 (4.2) | 1 (1.4) | 1 (1.4) |
| Teaching of medical students / trainees observing your clinics (n=45) | 0 (0.0) | 17 (37.8) | 25 (55.6) | 2 (4.4) | 0 (0.0) | 1 (2.2) |
| Teaching of AHPs / nurses (& AHP and nursing students) observing your clinics (n=68) | 1 (1.5) | 25 (36.7) | 36 (52.9) | 4 (5.9) | 1 (1.5) | 1 (1.5) |
| Formal teaching for medical staff / students / AHPs / nurses (n=30) | 0 (0.0) | 13 (43.3) | 13 (43.3) | 3 (10.1) | 1 (3.3) | 0 (0.0) |
| Supervision of less experienced rheumatology colleagues (n=55) | 0 (0.0) | 22 (40.0) | 29 (52.7) | 3 (5.5) | 0 | 1 (1.8) |
| Formal teaching of occupational therapists (n=33) | 0 (0.0) | 10 (30.3) | 18 (54.6) | 3 (9.1) | 1 (3.0) | 1 (3.0) |
| Lecturing for Higher Education Institutions (n=8) | 0 (0.0) | 1 (12.5) | 5 (62.5) | 1 (12.5) | 1 (12.5) | 0 (0.0) |
| Contribution to research (e.g., data collection, recruitment, intervention delivery) (n=40) | 0 (0.0) | 12 (30.0) | 22 (55.0) | 4 (10.0) | 1 (2.5) | 1 (2.5) |
| Leading of research projects and audits (n=24) | 0 (0.0) | 8 (33.3) | 13 (54.2) | 2 (8.3) | 1 (4.2) | 0 (0.0) |
| Other (n=7)* | 0 (0.0) | 1 (14.3) | 4 (57.1) | 0 (0.0) | 1 (14.3) | 1 (14.3) |

Legend: n, number; ADL, activities of daily living; conventional synthetic Disease-Modifying Anti-Rheumatic Drug; JAK, Janus kinase; DAS, disease activity score; MR, magnetic resonance; XR, x-ray; DEXA, Dual-Energy X-ray Absorptiometry; AHP, allied healthcare professional; IAPT, Improving Access to Psychological Therapies; * other activities such as: peer-mentoring, tailored exercises, supervision of junior physiotherapists, service development and workshop creation.
