# Supplementary material for: A scoping survey for the UK rheumatology occupational therapy capabilities framework
Source: Rheumatol Adv Pract. 2025 Jun 9;9(3):rkaf072. doi: 10.1093/rap/rkaf072 (PMC12202759; doi:10.1093/rap/rkaf072)
Supplement: rkaf072_Supplementary_Data [file rkaf072_supplementary_data.zip › 25-064 Supplementary Data S6.docx]

**Supplementary Data S6 - Self-Assessment of Comfort Level with EULAR Core Competencies in Day-to-Day Practice**

|  | n=88 (100) | | | | | |
| --- | --- | --- | --- | --- | --- | --- |
| **Statements, n(%)** | **Very Uncomfortable** | **Uncomfortable** | **Neutral** | **Comfortable** | **Very Comfortable** | **NA** |
| HPRs should have knowledge of the aetiology, pathophysiology, epidemiology, clinical features and diagnostic procedures of common RMDs, including their impact on all aspects of life | 1 (1.1) | 5 (5.7) | 9 (10.2) | 49 (55.7) | 24 (27.4) | 0 (0.0) |
| Using a structured assessment, HPRs should identify aspects that may influence individuals with RMDs and their families, including: (a) clinical characteristics, risks, red flags and comorbidities, (b) limits to their activity and participation and (c) personal and environmental factors | 1 (1.1) | 3 (3.4) | 13 (14.8) | 42 (47.7) | 29 (33.0) | 0 (0.0) |
| HPRs should communicate effectively: to make contributions to other healthcare providers and stakeholders in RMD care and to collaborate with other healthcare providers, signpost or refer where appropriate to optimise the interdisciplinary care of people with RMDs | 1 (1.1) | 1 (1.1) | 14 (15.9) | 34 (38.6) | 38 (43.2) | 0 (0.0) |
| HPRs should have an understanding of common pharmacological and surgical therapies in RMDs, including their anticipated benefits, side-effects and risks, and use this knowledge to advise or refer as appropriate | 2 (2.3) | 10 (11.4) | 19 (21.6) | 33 (37.5) | 24 (27.3) | 0 (0.0) |
| HPRs should provide advice on non-pharmacological interventions, treat or refer as appropriate, based on the evidence, expected benefits, limitations and risks for people with RMDs | 2 (2.3) | 1 (1.1) | 11 (12.5) | 33 (37.5) | 40 (45.5) | 1 (1.1) |
| HPRs should assess the educational needs of people with RMDs and their carers to provide tailored education using appropriate modes of delivery, relevant resources and evaluate their effectiveness | 2 (2.3) | 8 (9.1) | 16 (18.2) | 30 (34.1) | 32 (36.4) | 0 (0.0) |
| HPRs should take responsibility for their continuous learning and ongoing professional development to remain up-to-date with the clinical guidelines and/or recommendations on the management of RMDs | 1 (1.1) | 2 (2.3) | 7 (8.0) | 40 (46.0) | 35 (40.2) | 3 (3.4) |
| HPRs should support people with RMDs in goal setting and shared decision making about their care (e.g., identify, prioritise, address their needs and preferences and explain in lay terms) | 1 (1.1) | 1 (1.1) | 2 (2.3) | 35 (39.8) | 48 (54.5) | 1 (1.1) |
| HPRs should support people with RMDs in self-management of their condition. This encompasses selecting and applying the appropriate behavioural approaches and techniques to optimise their health and well-being (e.g., engagement in physical activity, pain and fatigue management) | 1 (1.1) | 1 (1.1) | 7 (8.0) | 33 (37.9) | 44 (50.6) | 2 (2.3) |
| HPRs should be able to select and apply outcome measures for people with RMDs, as appropriate, to evaluate the effectiveness of their interventions | 3 (3.4) | 5 (5.7) | 19 (21.8) | 31 (35.6) | 26 (29.9) | 4 (3.6) |

Legend: NA, Not Applicable; HPR, Health Professional in Rheumatology; RMD, Rheumatic and Musculoskeletal Diseases
